# Attitudes toward Family Planning among HIV-Positive Pregnant Women Enrolled in a Prevention of Mother-To-Child Transmission Study in Kisumu, Kenya

**DOI:** 10.1371/journal.pone.0066593

**Published:** 2013-08-26

**Authors:** Victor Akelo, Sonali Girde, Craig B. Borkowf, Frank Angira, Kevin Achola, Richard Lando, Lisa A. Mills, Timothy K. Thomas, Shirley Lee Lecher

**Affiliations:** 1 Kenya Medical Research Institute, Kisumu, Kenya; 2 Division of HIV/AIDS Prevention, U.S. Centers for Disease Control and Prevention, Atlanta, Georgia, United States of America; 3 ICF International, Atlanta, Georgia, United States of America; 4 U.S. Centers for Disease Control and Prevention, HIV Research Branch, Kisumu, Kenya; UCL Institute of Child Health, University College London, United Kingdom

## Abstract

**Background:**

Preventing unintended pregnancies among HIV-positive women through family planning (FP) reduces pregnancy-related morbidity and mortality, decreases the number of pediatric HIV infections, and has also proven to be a cost-effective way to prevent mother-to-child HIV transmission. A key element of a comprehensive HIV prevention agenda, aimed at avoiding unintended pregnancies, is recognizing the attitudes towards FP among HIV-positive women and their spouse or partner. In this study, we analyze FP attitudes among HIV-infected pregnant women enrolled in a PMTCT clinical trial in Western Kenya.

**Methods and Findings:**

Baseline data were collected on 522 HIV-positive pregnant women using structured questionnaires. Associations between demographic variables and the future intention to use FP were examined using Fisher's exact tests and permutation tests. Most participants (87%) indicated that they intended to use FP. However, only 8% indicated condoms as a preferred FP method, and 59% of current pregnancies were unintended. Factors associated with positive intentions to use FP were: marital status (p = 0.04), having talked to their spouse or partner about FP (p<0.001), perceived spouse or partner approval of FP (p<0.001), previous use of a FP method (p = 0.006), attitude toward the current pregnancy (p = 0.02), disclosure of a sexually transmitted infection (STI) diagnosis (p = 0.03) and ethnic group (p = 0.03).

**Conclusion:**

A significant gap exists between future FP intentions and current FP practices. Support and approval by the spouse or partner are key elements of FP intentions. Counseling services should be offered to both members of a couple to increase FP use, especially given the high number of unplanned pregnancies among HIV-positive women. Condoms should be promoted as part of a dual use method for HIV and STI prevention and for contraception. Integration of individual and couple FP services into routine HIV care, treatment and support services is needed in order to avoid unintended pregnancies and to prevent mother-to-child HIV transmission.

## Introduction

Women remain disproportionately affected by the HIV epidemic particularly in sub-Saharan Africa, where women comprise 58% of the adults living with HIV, according to the Joint United Nations Programme on HIV/AIDS (UNAIDS), 2012. In 2011, the incidence in children was also highest in sub-Saharan Africa, representing more than 90% of children worldwide who became newly infected with HIV [1]. Treatment and prevention of HIV infection in women is of course required to prevent new infections in infants. The number of HIV-infected children <15 years old in sub-Saharan Africa is 91% of the total 3.4 million HIV-infected children globally [1]. These unfavorable statistics, coupled with the high prevalence of HIV-infected pregnant women in sub-Saharan Africa, underscore the need for HIV interventions focused on prevention of mother-to-child transmission (PMTCT) and family planning (FP) to avoid unintended pregnancies [1]. It is estimated from data collected from 42 countries in sub-Saharan Africa that 14 million unintended pregnancies occur each year [2]. Among the 128 million women married or in a union who are 15 to 49 years old in sub-Saharan Africa, the unmet need for FP is estimated at 25% according to UNAIDS [3]. In resource-limited countries, too few women are receiving effective FP or HIV prevention and treatment services to protect themselves and their children [4].

For the elimination of mother-to-child transmission of HIV, the World Health Organization (WHO) recommends a comprehensive PMTCT strategy that includes: 1) primary prevention of HIV infection among women of childbearing age, 2) FP for preventing unintended pregnancies among HIV-infected women, 3) preventing HIV transmission from HIV-infected women to their infants and 4) treatment, care and support of HIV-infected women and their children [5]. The importance of FP has increasingly gained recognition as having a vital role in the prevention of HIV transmission. Reducing unintended pregnancies among HIV-positive women through FP reduces the number of HIV-infected infants as much as the use of antiretroviral (ARV) prophylaxis for PMTCT [6]–[8]. It also reduces the number of children potentially orphaned when parents die of an AIDS-related illness, and decreases the vulnerability of women and infants to morbidity and mortality related to pregnancy and lactation [9], [10]. In addition, FP substantially reduces the morbidity known to increase for HIV-infected women during the year following delivery [11]. Meeting the FP needs for women living with HIV contributes significantly to reducing the need for ARV prophylaxis and treatment [4]. FP has also proven to be a cost-effective strategy for prevention of HIV transmission [10], as contraception costs are less than those of PMTCT [12]. Projected FP use in Uganda, which has one of the highest fertility rates in the world, was estimated to potentially avert 21.6% of vertical HIV infections and 18.5% of pediatric deaths [7].

Despite these benefits, FP needs have remained unmet in areas of the world where the prevalence of HIV/AIDS is highest [13], [14]. Recent literature indicates the use of FP among HIV-infected women has remained low [15]–[17]. The use of FP is lower among women in HIV-1 heterosexual serodiscordant partnerships in East Africa compared to Southern Africa [18]. Demographic health survey data of HIV-infected women from Kenya and Malawi indicated nearly three-quarters did not want more children within the next 2 years or ever, but only 26% in Kenya and 19% in Malawi were using modern contraceptives [19]. In a study conducted to determine the usage of FP services among HIV-infected mothers in a PMTCT program at the Kitale District Hospital in Kenya [20], only 44% of the respondents were using some form of FP. These findings are consistent with a study done by Keogh, et al. [21] in Mwanza, Tanzania, which found low rates of contraceptive use among women attending antenatal clinics. In sub-Saharan Africa modern FP methods, which include hormonal contraception, condoms, IUDs and sterilization, are used with a higher prevalence overall than other methods, such as withdrawal and abstinence [16]. A prospective study of contraceptive use among African women from 7 countries, specifically Kenya, Rwanda, Tanzania, Uganda, Botswana, South Africa and Zambia, found that injectable contraceptives are the most common method used [18]. Other studies in Kenya, Uganda and Malawi have shown the most frequently used FP methods are hormonal (injectables, pills or implants) and condoms [18], [19], [22], [23].

There are several studies published on the knowledge and use of FP among HIV-infected individuals. There are, however, limited data on the attitudes of HIV-positive women towards the use of these FP methods, especially in Kenya [17], [22], [23]. Understanding the attitudes of HIV-positive women and their spouse or partner towards FP is critical to the expansion of comprehensive HIV prevention programs targeted at achieving a reduction in unwanted pregnancies and a decrease in the incidence of HIV-infected children.

The Kisumu Breastfeeding Study (KiBS) was a phase II open-label one-arm HIV PMTCT clinical trial conducted in Western Kenya. This clinical trial used a triple ARV regimen in the late antenatal period and during lactation until 6 months postpartum in order to reduce mother-to-child transmission. Questionnaires completed during enrollment collected information regarding whether the current pregnancy was intentional and whether participants anticipated using FP in the future. This current analysis using enrollment questionnaire data will help inform health care providers, policy makers and others involved in the development of FP services toward the creation and implementation of targeted FP strategies that specifically address the needs of HIV-positive women.

## Methods

### Study population

**Figure 1:**
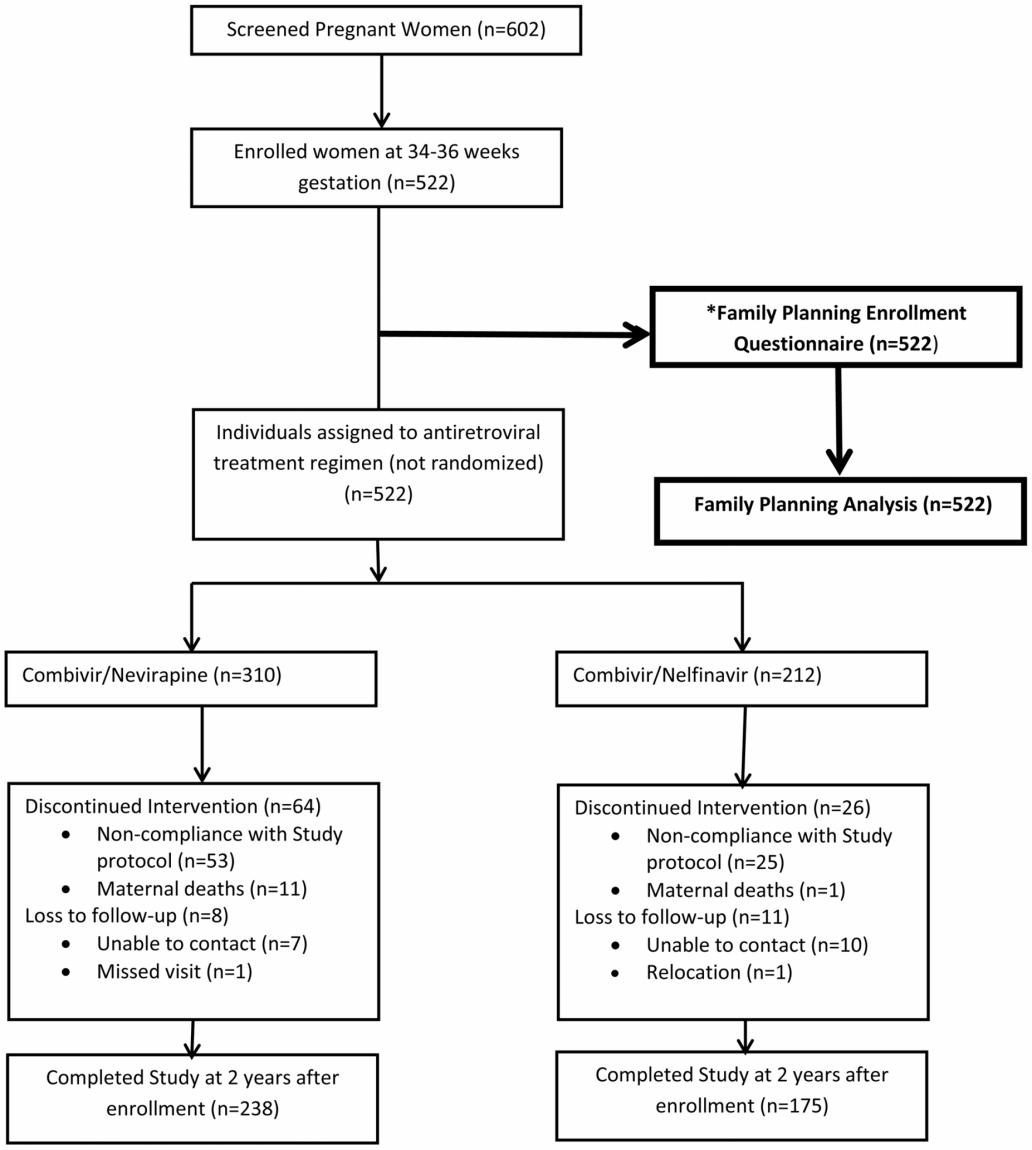
Flow diagram of women enrolled n the Kisumu breastfeeding study.

KiBS was conducted at the KEMRI-CDC Clinical Research Center, jointly operated by the Kenya Medical Research Institute (KEMRI) and the U.S. Centers for Disease Control and Prevention (CDC), located at the New Nyanza Provincial General Hospital (NNPGH) in Kisumu, Kenya. Between July 2003 and November 2006, 602 HIV-infected pregnant women were screened from the PMTCT antenatal clinics of two government hospitals, the NNPGH and the Kisumu District Hospital (KDH). Study procedures and primary findings for this PMTCT clinical trial have been published previously by Thomas, et al. [24]. The enrollment criteria included: age ≥15 years old, gestation of 34–36 weeks and no previous ARV exposure. Laboratory confirmation of HIV status was performed at screening before enrollment. There were 522 HIV-infected women enrolled (Figure 1). The questionnaire used for this analysis was administered at the time of enrollment. Over 60% of the women who delivered at NNPGH came from 3 neighborhoods in the lowest income areas of Kisumu. At both recruitment hospitals, all women were offered voluntary HIV counseling and testing as per the routine standard of care.

Participants provided written informed consent. The study was approved by the Ethical Review Committee of KEMRI and the Institutional Review Board of the U.S. CDC, Atlanta, GA, USA. For this analysis, the baseline data collected at enrollment included: maternal age, parity, level of education, whether current and future pregnancies were planned for and/or desired, prior knowledge and use of FP, future FP preferences, and perception of FP acceptance by their spouse or partner. Counseling on methods for preventing unintended pregnancies was offered at all study visits. Additional counseling was available to support the women as they managed the reaction of their spouse or partner to their decision about FP and condom use, as well as the disclosure of their HIV status.

### Data Collection

Data were collected using written study instruments with structured and semi-structured questions. Questionnaires were written in English and then translated into Dholuo and Kiswahili, which are the predominant languages of the area. Pilot testing and revision of questionnaires were completed prior to initiation of the study. Questionnaires were administered verbally by trained study nurse counselors.

### Data analysis

In the current analysis, descriptive statistics were used to describe baseline maternal characteristics and preferred FP methods. The term “family planning” includes contraceptive injectables, pills, implants, condoms, intrauterine devices, diaphragm, female/male sterilization, as well as other FP methods, such as abstinence, rhythm, withdrawal or a cultural/folk method. Fisher's exact test was used for two-by-two contingency tables and permutation tests for larger tables to test for associations between demographic and psychosocial variables and the intention to use FP methods. Statistical analyses were performed using SAS software, version 9.3 (SAS Institute, Inc., Cary, North Carolina, U.S.).

## Results

Data on demographics, psychosocial and behavioral factors were collected from 522 pregnant women at enrollment (Table 1). The median maternal age was 23 years (range 15–43 years) and the median gestational age at enrollment was 34 weeks. Most of the participants, 447 (86%), were from the Luo tribe, which is the fourth largest tribe in Kenya [25] and one of the main tribes in western Kenya. There were 392 (75%) women who had prior pregnancies before enrollment, with a median parity of 2, whereas 130 (25%) were primigravid. The majority of women, 388 (74%), were married, and 376 (72%) were living with the father of the current pregnancy. Only 69 (13%) were single and the remaining 65 (13%) were widowed, separated or divorced. Whereas 368 (70%) of the women had completed 8 years of education, 154 (30%) had not completed primary education. Importantly, only 186 (36%) of the women indicated that they desired the current pregnancy, 233 (45%) would have preferred to wait, and 74 (14%) did not want the pregnancy, indicating that 59% of pregnancies were unintended. The remaining 26 (5%) were either not sure or refused to answer the question.

**Table 1:** Baseline Maternal Characteristics of 522 HIV-Infected Women Initiating ARV Therapy for PMTCT in the Kisumu Breastfeeding Study, July 2003 to November 2006.*

Variable (n = 522)	Category	Number (%) or Median (Range)
Age (years)	15–19	76 (15%)
	20–24	221 (42%)
	25–29	142 (27%)
	≥30	83 (16%)
Median age (years)		23 (15–43)
Primigravid	Yes	130 (25%)
	No	392 (75%)
Median parity, among multigravid (n = 392)		2 (0–8)
Marital status	Single	69 (13%)
	Married	388 (74%)
	Separated/Divorced	26 (5%)
	Widowed	39 (8%)
Living with child's father (n = 521)	Yes	376 (72%)
	No	145 (28%)
Completed primary education (8 years)	Yes	368 (70%)
	No	154 (30%)
Employed outside home	Yes	174 (33%)
	No	348 (67%)
Median people in household (n = 521)		3 (1–14)
Level of income (KSh§/month) (n = 520)	<2,000	69 (13%)
	2,000–4,999	102 (20%)
	5,000–9,999	61 (12%)
	≥10,000	39 (8%)
	Unknown	249 (48%)
Ethnic group	Luo	447 (86%)
	Luhya	54 (10%)
	Other	21 (4%)
Attitude toward current pregnancy (n = 519)	Desired	186 (36%)
	Wanted to wait	233 (45%)
	Did not want to be pregnant	74 (14%)
	Unsure	23 (4%)
	Refused	3 (1%)

* Number indicates the number of women who chose each response. Percentages may not add up to 100% due to rounding. Sample sizes (in parentheses) may vary due to missing data.

§ KSh 85 per 1 US dollar (rate varies).

At baseline, the women were asked about their intention to use any form of FP in the future (Table 2). There were 448 (87%) women who indicated they intended to use FP, 44 (8%) did not intend to use FP and 25 (5%) did not know. Half of the women, 262 (50%), had discussed the issue of FP with their spouse or partner, whereas nearly the same number of women, 254 (49%), had not. When participants were asked to predict the feelings of their spouse or partner about the use of FP, 262 (50%) thought they would approve, whereas 122 (23%) thought they would not approve, and 135 (26%) responded that the feelings of their spouse or partner were unknown. In order to investigate perceptions of whether they shared a similar view on the desired number of children, we asked participants if they thought their spouse or partner wanted the same, more or fewer children than they did. There were 186 (36%) who reported their spouse or partner wanted the same number of children. By comparison, 90 (17%) reported their spouse or partner wanted more children, 25 (5%) stated they wanted fewer and 143 (27%) indicated they did not know the number of children their spouse or partner wanted.

**Table 2:** Women's Perception of Family Planning Acceptance by Spouse or Partner.*

Variable (n = 522)	Category	Number	Percent
Intention to use FP method in future (n = 517)	Intended to use	448	87%
	Did not intend	44	8%
	Unknown	25	5%
Have you ever talked with your spouse/partner about FP?	Yes	262	50%
	No	254	49%
	Unknown	4	1%
	Refused	2	<1%
Prediction of FP approval of by spouse/partner	Would approve	262	50%
	Would disapprove	122	23%
	Unknown	135	26%
	Refused	3	1%
Does your spouse/partner want the same number of children as you do? (n = 520)	Same	186	36%
	More	90	17%
	Fewer	25	5%
	Unknown	143	27%
	Refused	76	15%

* Number (percent) indicates the number (percentage) of women who chose each response. Percentages may not add up to 100% due to rounding. Sample sizes (in parentheses) may vary due to missing data.

In Table 3, we analyzed a number of variables to identify predictors of intention to use FP among the 492 participants from Table 2 who definitely stated they either intended to use or not to use FP. Among the 168 participants who desired the current pregnancy, 152 (90%) intended to use FP in the future. Of the 297 participants who responded that they either wanted to wait or did not want to be pregnant, 276 (93%) intended to use FP. Attitudes toward the current pregnancy were significantly associated with future intentions to use FP (p = 0.02). There was also a significant association between intent to use FP and having disclosed to their spouse or partner a history of a previous sexually transmitted infection (STI) other than HIV (p = 0.03), those who talked to their spouse or partner about the intention to use FP (p<0.001) and those whose spouse or partner approved of using FP (p<0.001). All of the women who answered the questionnaire indicated they had prior knowledge of FP. Previous use of FP was significantly associated with intention to use FP in the future (p = 0.006). FP also varied by marital status (p = 0.04). Specifically, married (93%) and single mothers (90%) reported higher rates than separated/divorced (83%) and widowed (82%) mothers (p = 0.02). Ethnic group was also associated with intention to use FP (p = 0.03). A higher percentage of the Luhya tribe (100%) expressed an intention to use FP compared with the Luo tribe (90%) and others (89%).

**Table 3:** Predictors of Intention to Use Family Planning.*

Variable (n = 492)	Category	Total	Number	Percent	P-Value
Age (years)	15–19	75	68	91%	0.39
	20–24	210	188	90%	
	25–29	131	119	91%	
	≥30	76	73	96%	
Parity	Primiparous	366	337	92%	0.20
	Multiparous	126	111	88%	
Marital status	Single	67	60	90%	0.04
	Married	363	337	93%	
	Separated/Divorced	24	20	83%	
	Widowed	38	31	82%	
Completed primary education (8 years)	Yes	347	319	92%	0.30
	No	145	129	89%	
Employed outside home	Yes	161	150	93%	0.31
	No	331	298	90%	
Number of sexual partners (n = 146)	0	70	59	84%	0.75
	1	55	49	89%	
	2–5	21	19	90%	
Level of income (KSh/month) (n = 490)	<2,000	66	59	89%	0.70
	2,000–4,999	100	89	89%	
	5,000–9,999	55	51	93%	
	≥10,000	34	33	97%	
	Unknown	235	214	91%	
Ethnic group	Luo	424	382	90%	0.03
	Luhya	49	49	100%	
	Other	19	17	89%	
Previous history of STI other than HIV (n = 489)	Yes	63	57	90%	0.81
	No	426	389	91%	
Disclosure of STI to spouse/partner§ (n = 63)	Disclosed	56	52	93%	0.03
	Did not disclose	7	4	57%	
Knowledge of FP (n = 482)	Yes	482	441	91%	NA
	No	0	0	NA	
Previously used a FP method (n = 457)	Yes	219	209	95%	0.006
	No	238	210	88%	
Attitude toward current pregnancy (n = 489)	Desired	168	152	90%	0.02
	Wanted to wait	228	209	92%	
	Did not want to be pregnant	69	67	97%	
	Unsure	21	16	76%	
	Refused	3	2	67%	
Talked to spouse/partner about FP (n = 490)	Yes	249	240	96%	<0.001
	No	237	204	86%	
	Unknown	4	3	75%	
Spouse/partner approval of FP use (n = 489)	Yes	248	241	97%	<0.001
	No	114	97	85%	
	Unknown	127	109	86%	
Concurrence in number of children with the spouse/partner (n = 490)	Same	180	168	93%	0.21
	More	83	77	93%	
	Fewer	24	23	96%	
	Unknown	132	118	89%	
	Refused	71	60	85%	

* Number (percent) indicates the number (percentage) of women of the total who intended to use family planning. Sample sizes (in parentheses) may vary due to missing data.

§ Only for 63 participants who had a previous history of an STI other than HIV.

We interviewed participants about their preferred FP methods. Choices were not mutually exclusive, as more than one option could be selected on the questionnaire. Approximately two-thirds (296) of the total respondents indicated that they preferred hormonal therapy (primarily injectable methods, such as Depo-Provera) among available FP methods (Table 4). Of note is the paucity of participants, only 36 (8%), who preferred condoms. To further examine condom use, we conducted an analysis looking at condom use and reasons for not using condoms. Nearly half, 243 (47%) of the 522 participants, had never used a condom. An analysis of women who had never used condoms (Table 5) revealed that 102 (43%) women reported their spouse or partner refused to use them. However, women did not cite lack of knowledge about where to access condoms or how to use them as reasons for failing to use condoms.

**Table 4:** Preferred Family Planning Method.*

Type	FP method	Total	Number	Percent
Hormonal	Injectables	474	207	44%
	Pills	474	54	11%
	Implants	474	35	7%
Barrier/Mechanical	Condom	474	36	8%
	IUD	472	7	1%
	Diaphragm	473	1	<1%
Behavioral	Periodic abstinence	474	19	4%
	Withdrawal	473	4	1%
Permanent	Female sterilization	474	60	13%
	Male Sterilization	474	1	<1%
Other FP Method		473	10	2%
Unsure about FP Method		470	55	12%

* Choices were not mutually exclusive. Number (percent) indicates the number (percentage) of women of the total who chose each FP method. Totals may vary due to missing data.

**Table 5:** Reasons Given by Women Who Had Never Used a Condom.*

Variable (n = 236)	Category	Number	Percent
Do not know where to get them (n = 235)	Yes	23	10%
	No	201	86%
	Unknown/Refused	11	5%
Do not know to use them (n = 235)	Yes	44	19%
	No	175	74%
	Unknown/Refused	16	7%
Spouse/partner refuses to use them	Yes	102	43%
	No	111	47%
	Unknown/Refused	23	10%
Church says not to use them	Yes	11	5%
	No	214	91%
	Unknown/Refused	11	5%
Condoms are laced with HIV	Yes	7	3%
	No	213	90%
	Unknown/Refused	16	7%
Too expensive	Yes	4	2%
	No	214	91%
	Unknown/Refused	18	8%

* Number (percent) indicates the number (percentage) of women who chose each response. Percentages may not add up to 100% due to rounding. Sample sizes (in parentheses) may vary due to missing data.

## Discussion

The majority (87%) of HIV-infected pregnant women participating in this PMTCT trial expressed an intention to use FP in the future. However, this contrasts with the high proportion of unintended pregnancies (59%), thus emphasizing a gap between intent and practice. Our findings are similar to those reported by other investigators in South Africa, Uganda and Rwanda [17], [22], [23]. An unintended pregnancy rate of 61.6% was found among HIV-positive postpartum women in Cape Town, South Africa [26]. Data from a large cohort study in Rakai, Uganda over the period 2000–2006 revealed nearly half of all pregnancies among HIV-infected women were unintended [27]. In a cross-sectional study among HIV-positive women on ART in Kigali, Rwanda, 62.7% had become pregnant unintentionally [28]. Such issues are not limited to sub-Saharan Africa, as similar findings were reported among HIV-positive women in Ontario, Canada, where 56% of HIV-positive women classified their last pregnancy as unintended [29]. In the United States a 2011 study reported 49% of all pregnancies were unintended [30], [31].

Modern FP methods (hormonal, barrier or permanent) were preferred by most of the women who intended to use FP. This preference reflects the FP counseling structure in Kenya, which puts more emphasis on the use of modern methods and the provision of such counseling services at the HIV care and treatment sites [32]. Although the data on FP was collected at the time of enrollment, participants may have had prior FP counseling since FP services are available within the Kenyan government hospitals, including NNPGH and KDH, which were used as recruitment sites for this trial. Counseling on the use of contraceptives was offered at all study visits during the trial. Nearly 60% of the women preferred hormonal contraceptives, with most of them preferring the injectable Depo-Provera. Hormonal contraceptives and condoms are easily accessible in Kenya government clinics and are provided at no cost. The Kenyan government provides 33–50% of hormonal contraceptives and condoms throughout the country [33]. The predominant preference toward hormonal contraceptives is consistent with other studies in Eastern Africa, Western Africa and South Africa [20], [22], [26], [27], [34]. In Rakai, Uganda, use of hormonal contraceptives tripled from 1994 to 2006, primarily by increased use of hormonal injectables and implants [27]. In Kenya, Bii, et al. also reported hormonal injectable contraceptives as the most popular method of FP [20]. In Tanzania, Keogh, et al. found HIV-positive women more likely to use hormonal methods of contraception [21]. Currently there is a controversy regarding the use of hormonal contraceptives and a possible increase in HIV transmission. A systematic review by Polis, et al. assessing various hormonal contraceptive methods found mixed evidence for an increased risk of HIV transmission [35]. An additional concern has been raised regarding the use of hormonal contraceptives and disease progression. A recent review of multiple cohort studies suggests that HIV-infected women can use hormonal contraceptives without concern for HIV disease progression [36].

Only 8% of participants chose condoms as a preferred FP method, similar to previous studies in Africa [21], [37]. In addition, other studies in Africa have also reported low condom use among sexually active HIV-positive individuals, ranging from 5.5% to 24% [26], [38]–[40]. Of the 522 participants in the study, 243 (47%) had never used a condom. Despite intensive efforts to encourage condom use and improve access in sub-Saharan Africa, the amount of condom usage remains suboptimal [39], [41]–[43]. There is a low acceptance of condom use in sub-Saharan Africa due to socio-cultural influences, gender and sexual norms, influences of poverty, and insufficient information [43]. Advocacy is needed not only for an increase in the availability of condoms in resource-limited settings, but also for an improvement in the awareness and acceptability of the role for condoms in HIV prevention and FP. Our findings and those of others highlight the need for improved and innovative counseling strategies and education on the benefits of condoms to decrease HIV transmission and to prevent the acquisition of STIs, in addition to FP. Among the factors identified for low condom preference, 43% (102) indicated refusal by the spouse or partner to use them. This highlights the pivotal role of spouse or partner support and negotiation because condom use requires partner cooperation and also emphasizes the potential role for more female-controlled methods of HIV prevention and contraception. Also important is the need for culturally integrative approaches when engaging male partners in interventional strategies.

Successful FP involves the participation of both the women and her spouse or partner. FP programs should be developed to target both men and women, recommending condoms in combination with other effective contraceptive methods. Dual use of condoms with other contraceptives is particularly important among discordant couples for averting HIV transmission [18], [44]. Nineteen percent (44) of the women who had never used a condom lacked knowledge about how to use them, although this information is generally available. Comprehensive services for HIV-infected individuals need to include informative and interactive discussions on the proper use of condoms and the dual protection that condoms provide so that individuals know how to protect themselves and others from disseminating HIV and acquiring other STIs. Another possible explanation for low condom use could be that the women had not disclosed their HIV status to their spouse or partner. Among HIV-positive women the condom acceptance rate as a FP method needs to be verified on a population basis in high HIV prevalence areas.

This study identified seven variables that were significantly associated with an increased intention to use FP: 1) marital status (p = 0.04), specifically being married or single vs. divorced, separated or widowed (p = 0.02), 2) having talked to the spouse or partner about FP use (p<0.001), 3) perceived spouse or partner approval of FP (p<0.001), 4) attitudes toward the current pregnancy (p = 0.02), previous use of a FP method (p = 0.006), 6) disclosure of a previous diagnosis of sexually transmitted infections (p = 0.03) and 7) ethnic group (p = 0.03). Being from the Luhya tribe vs. the Luo or other tribe was significant. The total percentage of Luhya who intended to use FP was 100% compared to 90% among the Luo or 89% for other tribes. Further investigation is needed to determine the underlying reasons for these differences. These results are consistent with the findings from other studies with a high percentage of unintended pregnancies in Uganda and another study in Kenya [23], [27], [45], which found a significant association of FP use with marital status, and having discussed FP with their partner. In addition to previous findings, our analysis uniquely revealed that the women's perceived spouse or partner approval of FP and disclosure of previous STIs were significantly associated with the intention to use FP in the future.

Three of the variables associated with intention to use FP, namely having talked to the spouse or partner about FP, spouse or partner approval of FP, and disclosure of sexually transmitted infections, reveal the importance of communication. These results suggest that open and effective communication with one's spouse or partner is more likely to result in use of a FP method. Communication, gender relationships and negotiating in decision making are important determinants of FP [46]. Having an agreement between both parties in the relationship is more likely to result in successful and sustained use of an effective FP method. These results underscore the importance of the role of the spouse or partner in the FP process. Whereas most men have limited FP knowledge [47], they remain crucial in the decision-making process on selection of FP options. Counseling for couples rather than individual counseling may prove more effective in promoting the uptake and use of FP options. There was a significant association between intention to use FP and whether the participant desired, wanted to wait, or did not want the current pregnancy. The high percentage of unintended pregnancies in our study, indicated by 59% overall (Table 1) and over 60% (Table 3) who indicated that they wanted to wait before having the current pregnancy or did not want to be pregnant, reflects a need to improve current FP strategies.

Health care professionals and public health personnel involved in patient care need to emphasize FP as a preventive measure to mitigate the spread of HIV infection. Integration of reproductive health services into voluntary HIV counseling and testing sites has resulted in increased use of contraceptives, reduced discontinuation of contraceptives and a significantly reduced pregnancy rate [48]. More innovative ways should be developed relevant to the cultural context to motivate uptake and encourage sustained use of contraceptives for women who do not desire to become pregnant [49]. FP and counseling services should be designed to target the unmet needs of HIV-positive women. This will increase the knowledge and awareness of options available for those who desire FP. The expected end result will be greater use of FP methods, yielding fewer unintended and unplanned pregnancies, thereby lowering the incidence of new HIV infections. There is a need for couple-oriented and male-oriented reproductive health services, in addition to addressing female reproductive health issues for HIV-positve women. Our findings highlight the need to incorporate FP services into routine HIV care and treatment services as part of a holistic HIV prevention approach.

### Limitations

Limitations of this study include recall bias on some of the questions that require the women to recall past information. Reporting information to an interviewer whom they may intend to please could result in a social desirability bias. The study participants were predominantly from an urban population in Kenya and thus the findings may not be generalizable to other settings. We could not measure the actual use of FP methods because this information was not collected. Also, condom use was only asked about in the context of contraceptive options and not for STI prevention. Finally, because HIV prevention and FP practices are changing over time, these data collected over the period 2003 to 2006 may differ from the most recent information at the time of this publication.

### Summary

Innovative approaches are needed to address the need for FP options available to HIV-positive women in resource-limited settings. A majority of these HIV-positive women in Western Kenya did not desire their current pregnancies and most were willing to use FP in the future. Unintended pregnancies among HIV-positive women represent an additional missed opportunity for prevention of mother-to-child transmission. Spouse or partner approval was crucial to a woman's intention to use FP, and therefore interactive discussions and agreement on FP methods are more likely to lead to successful implementation. FP and counseling services should be offered to both members of a couple to increase uptake, especially in the setting of a high number of unplanned pregnancies among HIV-positive women. Because condom use as a FP method in our study and many African countries is suboptimal, HIV prevention efforts should emphasize the importance of condoms as part of a dual use strategy for both HIV/STI prevention and as a contraceptive measure.

## References

[1] UNAIDS (2012) Report on the global AIDS epidemic 2012. Available: http://reliefweb.int/report/world/unaids-report-global-aids-epidemic-2012.

[2] Hubacher D, Mavranezouli I, McGinn E (2008) Unintended pregnancy in sub-Saharan Africa: magnitude of the problem and potential role of contraceptive implants to alleviate it. Contraception 78: 73–78. 18555821 10.1016/j.contraception.2008.03.002

[3] UNAIDS (2012) Women need access to dual protection-effective contraceptives and HIV prevention options. Available: wwwunaidsorg

[4] UNAIDS (2011) UNAIDS I 2011–2015 Strategy Getting to Zero.

[5] World Health Organization, Unicef AIDS (2007) Guidance on Global Scale-Up of The Prevention of Mother-To-Child Transmission of HIV towards Universal Access for Women, Infants and Young Children and Eliminating HIV and AIDS among Children. World Health Organization, The Interagency Task Team (IATT) on Prevention of HIV Infection in Pregnant Women, Mothers and their Children.

[6] Council P (2003) Population Council Annual Report.

[7] Hladik W, Stover J, Esiru G, Harper M, Tappero J (2009) The contribution of family planning towards the prevention of vertical HIV transmission in Uganda. PLoS One 4: e7691. 19888347 10.1371/journal.pone.0007691 PMC2766039

[8] Reynolds HW, Steiner MJ, Cates W Jr (2005) Contraception's proved potential to fight HIV. Sex Transm Infect 81: 184–185. 15800107 10.1136/sti.2004.012013 PMC1764682

[9] Rutenberg N, Baek C (2005) Field experiences integrating family planning into programs to prevent mother-to-child transmission of HIV. Stud Fam Plann 36: 235–245. 16209180 10.1111/j.1728-4465.2005.00064.x

[10] Halperin DT, Stover J, Reynolds HW (2009) Benefits and costs of expanding access to family planning programs to women living with HIV. AIDS 23 Suppl 1: S123–130. 20081384 10.1097/01.aids.0000363785.73450.5a

[11] Chersich MF, Luchters SM, Yard E, Othigo JM, Kley N, et al (2008) Morbidity in the first year postpartum among HIV-infected women in Kenya. Int J Gynaecol Obstet 100: 45–51. 17900585 10.1016/j.ijgo.2007.06.053

[12] Reynolds HW, Janowitz B, Wilcher R, Cates W (2008) Contraception to prevent HIV-positive births: current contribution and potential cost savings in PEPFAR countries. Sex Transm Infect 84 Suppl 2: ii49–53. 18799493 10.1136/sti.2008.030049

[13] USAID (2006) ISSUE BRIEF Adding Family Planning to PMTCT Sites Increases PMTCT Benefits.

[14] United Nations Department of Economic and Social Affairs PD (2011) 2011 Update for the MDG Database: Unmet Need or Family Planning. Available: www.unorg/esa/population/publications/2011-mdgdatabase/2011_Update_MDG_UMNxls

[15] Heys J, Kipp W, Jhangri GS, Alibhai A, Rubaale T (2009) Fertility desires and infection with the HIV: results from a survey in rural Uganda. AIDS 23 Suppl 1: S37–45. 20081387 10.1097/01.aids.0000363776.76129.fd

[16] Cleland JBS, Ezeh A, Faundes A, Glasier A, Innis J (2006) Family planning: the unfinished agenda. Lancet 368: 1810–1827. 17113431 10.1016/S0140-6736(06)69480-4

[17] Homsy J, Bunnell R, Moore D, King R, Malamba S, et al (2009) Reproductive intentions and outcomes among women on antiretroviral therapy in rural Uganda: a prospective cohort study. PLoS One 4: e4149. 19129911 10.1371/journal.pone.0004149 PMC2612743

[18] Heffron R, Were E, Celum C, Mugo N, Ngure K, et al (2010) A prospective study of contraceptive use among African women in HIV-1 serodiscordant partnerships. Sex Transm Dis 37: 621–628. 20601930 10.1097/OLQ.0b013e3181e1a162

[19] Anand A, Shiraishi RW, Bunnell RE, Jacobs K, Solehdin N, et al (2009) Knowledge of HIV status, sexual risk behaviors and contraceptive need among people living with HIV in Kenya and Malawi. AIDS 23: 1565–1573. 19542867 10.1097/QAD.0b013e32832cb10c

[20] Bii SC, Otieno-Nyunya B, Siika A, Rotich JK (2008) Family planning and safer sex practices among HIV infected women receiving prevention of mother-to-child transmission services at Kitale District Hospital. East Afr Med J 85: 46–50. 18543527 10.4314/eamj.v85i1.9606

[21] Keogh SC, Urassa M, Kumogola Y, Mngara J, Zaba B (2009) Reproductive behaviour and HIV status of antenatal clients in northern Tanzania: opportunities for family planning and preventing mother-to-child transmission integration. AIDS 23 Suppl 1: S27–35. 20081386 10.1097/01.aids.0000363775.68505.f1

[22] Nattabi B, Li J, Thompson SC, Orach CG, Earnest J (2011) Family planning among people living with HIV in post-conflict Northern Uganda: A mixed methods study. Confl Health 5: 18. 21933403 10.1186/1752-1505-5-18 PMC3191370

[23] Wanyenze RK, Tumwesigye NM, Kindyomunda R, Beyeza-Kashesya J, Atuyambe L, et al (2011) Uptake of family planning methods and unplanned pregnancies among HIV-infected individuals: a cross-sectional survey among clients at HIV clinics in Uganda. J Int AIDS Soc 14: 35. 21718524 10.1186/1758-2652-14-35 PMC3136398

[24] Thomas TK, Masaba R, Borkowf CB, Ndivo R, Zeh C, et al (2011) Triple-antiretroviral prophylaxis to prevent mother-to-child HIV transmission through breastfeeding–the Kisumu Breastfeeding Study, Kenya: a clinical trial. PLoS Med 8: e1001015. 21468300 10.1371/journal.pmed.1001015 PMC3066129

[25] Kenya Go (2009) Kenya 2009 Population and Housing Census.

[26] Crede S, Hoke T, Constant D, Green MS, Moodley J, et al (2012) Factors impacting knowledge and use of long acting and permanent contraceptive methods by postpartum HIV positive and negative women in Cape Town, South Africa: a cross-sectional study. BMC Public Health 12: 197. 22424141 10.1186/1471-2458-12-197 PMC3328250

[27] Polis CB, Gray RH, Lutalo T, Nalugoda F, Kagaayi J, et al (2011) Trends and correlates of hormonal contraceptive use among HIV-infected women in Rakai, Uganda, 1994–2006. Contraception 83: 549–555. 21570553 10.1016/j.contraception.2010.10.002

[28] Kikuchi K, Wakasugi N, Poudel KC, Sakisaka K, Jimba M (2011) High rate of unintended pregnancies after knowing of HIV infection among HIV positive women under antiretroviral treatment in Kigali, Rwanda. Biosci Trends 5: 255–263. 22281539 10.5582/bst.2011.v5.6.255

[29] Loutfy M, Raboud J, Wong J, Yudin M, Diong C, et al (2012) High prevalence of unintended pregnancies in HIV-positive women of reproductive age in Ontario, Canada: a retrospective study. HIV Med 13: 107–117. 22103297 10.1111/j.1468-1293.2011.00946.x

[30] Centers for Disease Control and Prevention DoRH (2012) Unintended Pregnancy Prevention Available: www.cdcgov/reproductivehealth/unintendedpregnancy/.

[31] Finer LB, Zolna MR (2011) Unintended pregnancy in the United States: incidence and disparities, 2006. Contraception 84: 478–485. 22018121 10.1016/j.contraception.2011.07.013 PMC3338192

[32] Kosgei RJ, Lubano KM, Shen C, Wools-Kaloustian KK, Musick BS, et al (2011) Impact of integrated family planning and HIV care services on contraceptive use and pregnancy outcomes: a retrospective cohort study. J Acquir Immune Defic Syndr 58: e121–126. 21963940 10.1097/QAI.0b013e318237ca80 PMC3779789

[33] Division of Primary Health Care Ministry of Health Government of Kenya (October 2000) Family Planning and Reproductive Health Commodities in Kenya. Background Information for Policymakers Available: http://www.rti.org/pubs/Kenya_Family_Planning.pdf.

[34] Kibuuka H, Guwatudde D, Kimutai R, Maganga L, Maboko L, et al (2009) Contraceptive use in women enrolled into preventive HIV vaccine trials: experience from a phase I/II trial in East Africa. PLoS One 4: e5164. 19360102 10.1371/journal.pone.0005164 PMC2664465

[35] Polis CB, Phillips SJ, Curtis KM (2013) Hormonal contraceptive use and female-to-male HIV transmission: a systematic review of the epidemiologic evidence. AIDS 27: 493–505. 23079808 10.1097/QAD.0b013e32835ad539

[36] Phillips SJ, Curtis KM, Polis CB (2012) Effect of hormonal contraceptive methods on HIV disease progression: a Systematic Review. AIDS 10.1097/QAD.0b013e32835bb672 23135169

[37] Ngubane N, Patel D, Newell ML, Coovadia HM, Rollins N, et al (2008) Messages about dual contraception in areas of high HIV prevalence are not heeded. S Afr Med J 98: 209–212. 18350224

[38] Beyeza-Kashesya J, Kaharuza F, Ekstrom AM, Neema S, Kulane A, et al (2011) To use or not to use a condom: a prospective cohort study comparing contraceptive practices among HIV-infected and HIV-negative youth in Uganda. BMC Infect Dis 11: 144. 21605418 10.1186/1471-2334-11-144 PMC3128049

[39] Maharaj P, Neema S, Cleland J, Busza J, Shah I (2012) Condom use within marriage: an assessment of changes in South Africa and Uganda. AIDS Care 24: 444–450. 22085286 10.1080/09540121.2011.613913

[40] Subramanian L, McGrath N, Ndlovu H, Gafos M (2008) Family planning methods among women in a vaginal microbicide feasibility study in rural KwaZulu-Natal, South Africa. Afr J Reprod Health 12: 45–63. 20695041

[41] Cleland J, Ali MM (2006) Sexual abstinence, contraception, and condom use by young African women: a secondary analysis of survey data. Lancet 368: 1788–1793. 17113428 10.1016/S0140-6736(06)69738-9

[42] Wand H, Ramjee G (2012) The effects of injectable hormonal contraceptives on HIV seroconversion and on sexually transmitted infections. AIDS 26: 375–380. 22156970 10.1097/QAD.0b013e32834f990f

[43] Maticka-Tyndale E (2012) Condoms in sub-Saharan Africa. Sex Health 9: 59–72. 22348634 10.1071/SH11033

[44] Higgins JA, Cooper AD (2012) Dual use of condoms and contraceptives in the USA. Sex Health 9: 73–80. 22348635 10.1071/SH11004

[45] Mutiso SM, Kinuthia J, Qureshi Z (2008) Contraceptive use among HIV infected women attending Comprehensive Care Centre. East Afr Med J 85: 171–177. 18700350 10.4314/eamj.v85i4.9641

[46] Montgomery CM, Lees S, Stadler J, Morar NS, Ssali A, et al (2008) The role of partnership dynamics in determining the acceptability of condoms and microbicides. AIDS Care 20: 733–740. 18576176 10.1080/09540120701693974

[47] King R, Khana K, Nakayiwa S, Katuntu D, Homsy J, et al (2011) ‘Pregnancy comes accidentally–like it did with me’: reproductive decisions among women on ART and their partners in rural Uganda. BMC Public Health 11: 530. 21726467 10.1186/1471-2458-11-530 PMC3223906

[48] Duerr A, Hurst S, Kourtis AP, Rutenberg N, Jamieson DJ (2005) Integrating family planning and prevention of mother-to-child HIV transmission in resource-limited settings. Lancet 366: 261–263. 16023518 10.1016/S0140-6736(05)66917-6

[49] Ngure K, Heffron R, Mugo NR, Celum C, Cohen CR, et al (2012) Contraceptive method and pregnancy incidence among women in HIV-1-serodiscordant partnerships. AIDS 26: 513–518. 22156966 10.1097/QAD.0b013e32834f981c PMC3932300

